# Foreign Summary

**Published:** 1839-10-26

**Authors:** 


### FOREIGN SUMMARY.

### Velpeau's Clinical Lectures on Ophthalmia. No. IV.

*Inflammatory Diseases of the Globe of the Eye.*

We are now about to commence the study of the department of pathology which deserves more especially the name of ophthalmology. The various inflammatory affections of the palpebræ might justly be treated apart from the diseases of the eye, as these organs are merely the "tutamina oculi," destined to protect it from the action of external agents. Thinking it, however, better to follow the course usually pursued by authors, when treating of this subject, I have, in my former lectures, given an account of the different forms of blepharitis. I shall now proceed to examine the inflammatory affections of the conjunctiva, the cornea, and the iris. I also intend making a few observations on what has been called *sclerotitis.* These diseases I shall consider as purely inflammatory, as existing without any complication, and entirely independent of specific causes, reserving until a latter period the examination of the influence which these causes may exercise on ophthalmia in general. By thus gradually proceeding from what is simple to what is compound, our ideas will necessarily be more clear and more precise than were we to follow any other method.

The characters which serve to distinguish the inflammatory disease we are about to examine, are drawn, in a great measure, from the vascular appearance of the affected tissues. The importance of this symptom has been so generally admitted that in these later times many anatomical researches have been undertaken, with the view of throwing additional light on the distribution of the vessels of the eye. As I think it indispensable that you should be acquainted with the results of these researches, I shall enter into a few details on the subject.

Nearly all the arteries of the eye derive their origin from the ophthalmic branch given off from the internal carotid. The divisions of this artery, you are well aware, do not all follow the same direction, or terminate in the same tissues. Viewed in this light, they may be divided into four sets. Some supply the eyelids, others the conjunctiva; some, again, are distributed to the sclerotica, whilst others pass into the interior of the eye. These groups anastomose freely with one another.

The arterial network of the eyelids is principally formed by three branches—the nasal, the lacrymal, and the frontal arteries. These arteries, however, are only distributed to the mucous surface of the palpebræ, and to their free margin; the cutaneous surface is supplied by the temporal, the infra-orbital, the transversal, and angularis faciei. Thus the arterial circulation is carried on by two different sets of vessels, which anatomical fact may account for our meeting separately with the different forms of blepharitis.

The conjunctiva also presents two distinct sets of arteries. Some of these are given off by the branches we have seen supplying the mucous surface of the palpebræ, the remainder are ramifications of the superior and inferior muscular branches. These arteries are exceedingly numerous ; they may be seen presenting the appearance of tortuous, arborescent filaments, frequently anastomosing with one another, moveable at the centre of the ocular conjunctiva, immoveable near the cornea and near the fixed margin of the tarsal cartilages. Near the cornea, they communicate with the arteries of the sclerotica, and with those of the interior of the eye ; near the tarsal cartilages, with the arteries of the external surface of the eyelids. In some cases of intense simple conjunctivitis, these small vessels may be seen extending more or less on the cornea.

The sclerotica receives but a small number of arteries, nearly all of which are supplied from the muscular branches. These vessels present but few ramifications ; they communicate, in their course, with the arteries of the conjunctiva and those of the interior of the eye, and at their termination contribute to form the vascular circle round the cornea, which we are about to examine.

The ciliary branches are the only arteries supplying the interior of the eye, to which I shall direct your attention ; their mode of termination, on arriving at the ciliary circle, should be attentively studied. Some of these arteries pass outwards, and then, becoming reflected, anastomose with those of the conjunctiva and sclerotica ;—others, passing inwards, supply the iris, whilst a few, following their primitive direction, reach the cornea. The circumference of the cornea may, therefore, be termed the conflux of the arteries of the eye, as it is the seat of the vascular communication which exists between the exterior and the interior of that organ. The existence of this kind of external conflux bears so directly on the pathology of the eye, that it is indispensable you should be perfectly acquainted with the arteries by which it is formed.

The venous circulation of the eye is of but little importance with regard to the study of ophthalmology. The distribution of the veins, moreover, being nearly the same as that of the arteries, I shall not detain your attention any longer on this subject, but enter at once into the examination of the inflammatory affections of the eye, beginning by those of the external or mucous surface.

*Conjunctivitis.*

The ocular and palpebral conjunctiva being formed of the same anatomical elements, are consequently subject to the same forms of inflammation, with the exception of those inflammatory affections which attack the free margin of the palpebræ. The symptoms, however, are much more decided, much easier recognised, when the mucous membrane of the eye is inflamed, than when that of the palpebræ is affected.

The division we adopted for blepharitis will, therefore, in a great measure, be applicable to conjunctivitis or inflammation of the ocular conjunctiva. The affections which this general term comprise are the following :—

1. Simple Conjunctivitis.
2. Conjunctivitis with Chemosis.
3. Papular Conjunctivitis.
4. Granular Conjunctivitis.
5. Purulent Conjunctivitis.

This division is not arbitrary, as some persons might feel inclined to suppose ; it is founded on an attentive and lengthened observation of disease, and you will see that its utiliy is not merely theoretical when we arrive at the treatment of conjunctivitis. Those of you who follow the practice of this hospital, must be well aware that these forms of disease are frequently met with ; indeed we have now in our wards patients on whom you may observe each of the affections I have mentioned. I will, in the first place, describe the anatomical and physiological symptoms of each form of inflammation, and then examine at length the various agents we can employ in their treatment.

*Simple Conjunctivitis.*

This form of inflammation—the mildest we shall have to examine—is also the most frequent. The anatomical characters by which we may recognise it are the following :—the conjunctiva becomes of a deep or pale red colour ; the redness, however, is not constant, being sometimes modified by a yellow, purple, violet, or brick-red tinge. On the surface of the eye we distinguish a great number of small vessels of variable calibre, interlaced in many directions. Their mobility—the ease with which they are displaced, on pressing the eye finger, through the medium of the lower eyelid—show plainly that they belong to the conjunctiva. The nearer to the cornea you examine these vessels, the smaller and the less moveable you find them. They either terminate imperceptibly near the circumference of that membrane, or, becoming inflected, anastomose with the vessels of the eye. When the inflammation is intense, we sometimes see a few arterial filaments, reaching the cornea, advance more or less on its surface. The white colour of the sclerotica is still easily perceived through the injection of the conjunctiva.

To these, the anatomical symptoms, we must add those which are entirely functional. The secretion of mucus, which is much increased, varies considerably in its physical characters ; in some instances, clear, limpid and transparent, it flows, more or less abundantly over the eyelids, often producing an eczema of the parts over which it passes ; in others, on the contrary, it is opaque, and being detained by the cilia during sleep, concretes on the free margin of the eyelid, so that when the patients awake, they find their eyelids glued together. The mucus, also sometimes accumulates in the inner angle of the eye.

The secretion may be entirely suppressed, in which case the mucous membrane becomes dry, and presents a shining appearance. This symptom deserves particular attention, as there is reason to fear its being the commencement of a very serious disease, *xerophthalmia**. A circumstance which is also worthy of remark is, that in simple conjunctivitis we meet with neither photophobia nor shedding of tears. Several ophthalmologists of merit, M. Jungken for instance, have asserted that these symptoms are generally present; their opinions, however, on this subject, are incorrect, and must have been founded on cases presenting some other lesion. The visual functions are not in the least disordered in simple conjunctivitis, or indeed, in any of the forms of inflammation we are now examining, unless there be chemosis to such an extent as nearly to conceal the cornea. This circumstance should be kept in mind, as it may be extremely useful in establishing the diagnosis. The pain felt by the patient is slight, and seems merely to consist in the peculiar sensation of an extraneous body on the surface of the eye, which we have already met with in mucous blepharitis.

*Conjunctivitis with Chemosis.*

When the inflammation runs high, the symptoms we have just described become more intense, and chemosis sometimes supervenes, in which case the eye assumes a most peculiar appearance. The conjunctiva is uniformly of a deep violet colour, and the injected vessels which give rise to the vascularization are no longer to be distinguished from one another. The tissue of the conjunctiva is considerably thickened, and the cellular layers on its internal surface, which separate it from the sclerotica, become infiltrated with blood, so that the white colour of the sclerotica is completely hidden from view. The surface of the eye, of a fungous consistence, and a livid hue, presents a peculiar dotted appearance, similar to what we meet with in the cerebral substance after congestion of the brain. The conjunctiva thus tumefied, thus thickened, more or less elevated above the usual level of the globe of the eye, forms a complete circle round the cornea; indeed the chemosis is sometimes so great as to cover a great portion of the cornea, and consequently to greatly impair the visual functions of the eye. Chemosis is not only interesting as a morbid symptom, but also in an anatomical point of view; for although the limitation of the swelling of conjunctiva does not incontestably prove that the mucous membrane of the eye terminates at the circumference of the cornea, it nevertheless establishes, as an undeniable fact, that in this region it becomes extremely adherent to the subjacent tissues.

This, the inflammatory form of chemosis, may be compared, in many respects, to the phlegmonous erysipelas of other parts of the body, and might be termed the phlegmonous variety of chemosis—the tumefaction and swelling of the conjunctiva, which constitute this disease, not being always the result of violent inflammation, as most authors have asserted. It is, indeed, now generally admitted that chemosis is not, properly speaking, a disease, but merely a morbid symptom, generally the result of intense inflammation, sometimes, however, to be attributed to altogether different causes. The subconjunctival cellular layers may become infiltrated with serosity, and that not only in persons of a lymphatic constitution, but also in those who are young and robust, although the inflammation has been but very slight. The mucous membrane becomes tumefied by the accumulation of a serous fluid in the lamellæ of its tissue, without, however, presenting the characters I have described as accompanying the inflammatory form. The colour, instead of being of a dark-red or violet hue, is white, inclining to yellow; and the swollen tissues do not present the tense, elastic, turgid appearance we before noticed. The conjunctiva may, it is true, become considerably tumefied; but its tissue is flaccid, partly retaining the impression of the finger. In the phlegmonous form of chemosis there is often violent pain, caused by the compression of the eye by the chemosis; in this, the serous or œdematous form, there is, on the contrary, little, or no pain.

These two forms of chemosis are evidently distinct, and ought not to be confounded; you will now, however, I think, be able at once to distinguish them.

*Partial Conjunctivitis.*

The inflammation does not always attack the entire ocular conjunctiva; it may occupy a portion only of its surface. When this, the partial form of conjunctivitis, is not caused by external injury, it is generally to be met with near one of the angles of the eye, more especially near the external angle, and has consequently been called angular conjunctivitis. The inflamed surface is more or less circumscribed, and forms a kind of triangle, the basis of which is turned towards the the cornea, and the summit towards the angle of the side affected. The appearance it presents might not inaptly be compared to that of a slight ecchymosis, presenting small moveable tortuous vessels, some of which extend beyond the limits of the inflammation. I shall not enter into any further detail respecting this affection, as with the exception of the peculiar appearance, it is in every other respect similar to simple conjunctivitis.

*Papular Conjunctivitis.*

Accompanying inflammation of the conjunctiva, we sometimes observe on the mucous surface a kind of small papula, which has been generally confounded with ulceration of that membrane.

* In the 16th number of "La Presse Medicale," Feb. 1837, will be found an interesting article, by M. Jeanulme, on this affection, the cuticular conjunctiva of Mr. Travers. M. J. gives a detailed account of several cases which have occurred in the wards of MM. Velpeau and Sanson, and has thus thrown additional light on the nature of a disease to which pathologists have hitherto paid but little attention.

We have now in our wards several patients presenting this form of the disease, and we have frequently cases of a similar nature. Were you to examine them attentively, you would find that the abnormal appearance is not to be attributed to the presence of ulceration, but is occasioned by small aphthæ, analogous to those we meet with on the mucous membrane of the mouth. The mucous tissue which covers these papulæ would necessarily be destroyed were ulceration to exist; this, however, is not the case; there is no ulcerated surface, but merely small circumscribed tumefaction of the conjunctiva, which, by the friction they exercise on the internal surface of the eyelids, give rise to the peculiar sensation I have so frequently alluded to—that of an extraneous body between the eye and the eyelid—and are, as long as they remain, a source of continual irritation to the eye.

*Granular Conjunctivitis.*

Most of the characters which I gave when speaking of granular blepharitis, may be applied to granular inflammation of the conjunctiva.—There are, however, some few symptoms peculiar to this affection, which I must not omit to mention. The inflammation of the mucous follicles—for they are evidently the seat of the disease—may be either acute or chronic. It is impossible to deny the existence of the acute form of this disease, although the chronic is by far the most frequent. We have had, indeed, lately, several patients in our wards presenting the former or acute form of inflammation; and many of you, no doubt, will remember perfectly well having seen them; and why, I ask, should not the follicles of the mucous membrane of the eye become primitively inflamed, as well as the follicles of other tissues of a similar nature?

The more prominent characters by which we may recognise this affection are the following:—The mucous surface of the conjunctiva is of a paler red, and the vessels are less distinct, than in simple conjunctivitis; the colour is also, in most instances, uniformly the same. On examining minutely the conjunctiva, we find it covered with an immense number of granulations, of variable size, which are sometimes congregated together, as it were, on one portion of the mucous membrane, sometimes more or less separated from one another. When the disease persists, the conjunctiva assumes a velvety appearance. As in granular blepharitis, the mucous secretion varies in its physical properties. Sometimes it is increased, and then it may be limpid, opaque, and thick, or purulent; sometimes, on the contrary, the secretion is diminished, or even entirely suppressed; and should this suppression continue during any length of time, we might fear its being a symptom of incipient xerophthalmia. There is neither photophobia nor shedding of tears, these two symptoms, as I have already remarked, being absent in every form of conjunctival inflammation, unless accompanied by keratitis or iritis. The visual functions are not at all disordered. The sensation of foreign bodies on the surface of the eye is, as might be expected, extremely well marked.

This form of conjunctivitis appears identical with the disease described by German authors under the name of *catarrhal ophthalmia*, although many of the symptoms they mention are common to the various forms of conjunctivitis we have already examined. I shall not, however, discuss this question now, as we have agreed to defer the examination of "specific affections" until a later period.

Such are the different forms of conjunctivitis, which attentive observation enables us to distinguish. Generally speaking, these affections are combined with one another; indeed, the cases in which you find them isolated are comparatively rare, as we have already seen it to be the case with the various forms of blepharitis.

I have yet to speak of purulent conjunctivitis, to complete the description of the inflammatory affections of the mucous membrane of the eye. As, however, the only resemblance between this disease and those we have just examined is in the seat of the inflammation being the same in both cases, I will first give you the treatment of the different forms of conjunctivitis we have passed in review.—*Lond. Med. Gaz.*

---

### Velpeau's Clinical Lectures on Ophthalmia. No. VII.

### INFLAMMATORY AFFECTIONS OF THE CORNEA.

*Coloration of the Cornea.*

Returning to the consideration of the anatomical symptoms of keratitis, we must now study the changes which take place in the coloration of the cornea, when it has become the seat of inflammation. When examined in the different stages of the disease, it will be found that the cornea may be of four distinct shades or tints; with these it is important that you should become acquainted, as they will greatly assist you in the diagnosis of the various forms of inflammation we shall have to study.

*The water-green tint.*—The shade to which this term is applied is met with during the first period of the inflammation, and is not easily distinguished at first sight. Indeed, to form a correct estimate of the change that has taken place, the cornea must be attentively examined, and that on a patient who has only one eye affected. I describe the appearance which it then presents, under the name of the water-green tint, from the likeness which exists between the colour of the cornea and that of a sheet of water spread over a large surface, the transparency of which it very faithfully represents. Viewed sideways, and partially shaded from the light, the cornea has also a peculiarly moist humid appearance. These phenonomena are not, in my opinion, to be attributed to any change in the tissue of the cornea itself, but to a change in the aqueous humour of the anterior chamber. I do not, however, attach much importance to this explanation.

*The brown tint.*—When the inflammation continues, the cornea soon becomes of a light brown colour. On examining it attentively, we also generally find that it is covered, either entirely or partially, with a great number of exceedingly small granulations, smaller even than those which I described as existing in granular conjunctivitis. These granulations give the surface of the cornea a rough and uneven appearance, which we might aptly compare to that of a mucous membrane in the healthy state, and constitute, no doubt, what has been described by M. Lepelletier under the name of granular ophthalmia. The change which takes place in the colour of the membrane evidently depends on an alteration of its tissue—an alteration which may be referred to the external lamellæ. We shall also see presently that it is a symptom of superficial keratitis.

*The yellow tint.*—A yellow coloration of the cornea is a far more serious symptom than any we have yet examined, experience having shown that it always indicates a deep-seated affection of the tissue of the organ. The yellow tint first appears as a speck in the centre of the cornea, or as a narrow band or circle round its circumference. This circle may be complete or incomplete; in the latter case it is generally found to occupy the inferior portion of the cornea. It is important that you should become familiar with this symptom, as you might otherwise mistake it for a natural appearance caused by the reception of the cornea within the anterior border of the sclerotica. Such an error would probably be attended with disastrous consequences, the rapidity with which the yellow coloration extends being such, that, unless active measures are adopted to arrest its progress, the entire cornea is invaded, and vision is nearly always more or less injured.

*The dark red tint.*—This epithet does not convey as clear a notion of the colour which it is intended to describe as the expression adopted by Mr. Wardrop. He calls it the flint-stone colour; and those of you who have paid attention to diseases of the cornea, and have been able to observe it in the state the word represents, must confess that it is strictly appropriate. The change in the colour of the cornea generally commences, as with the yellow tint, in the centre.

*Suffusion of the Cornea.*

Besides the changes in colour which the cornea undergoes, when inflamed, it may also become the seat of suffusion, which constitutes the slightest form of opacity. When this is the case, the transparency of the cornea appears rather dimmed. It might be compared to the surface of polished marble over which the finger has been passed, or to a mirror on which a person has breathed, and which is still obscured by a slight vapour. If this suffusion, this haziness, of the cornea increases, it takes the name of *nebula*, ophthalmologists having fancied that a resemblance exists between the slight opacities which are then observed, and the airy clouds we see on a fine day floating in the air, like flakes of snow, festooned by the rays of the sun. The transparency of the cornea becomes more and more impaired when the inflammation does not abate; as, however, a considerable degree of suffusion is generally accompanied by ulceration of the cornea—a lesion which I intend to examine separately—we will not, for the present, pursue any further the examination of this symptom.

Now that we have studied the anatomical characters of keratitis—those which the Germans call the objective—we must direct our attention to the physiological or subjective symptoms of the disease. Although I have used the terms objective and subjective, I must tell you that I by no means admire them; they ought not, indeed, in my opinion, to be employed, as the words anatomical and physiological, or functional, are decidedly preferable. It is well, however, that you should be acquainted with their signification, as they are frequently used by German writers.

The physiological symptoms of keratitis are—intolerance of light, or photophobia; shedding of tears, or epiphora; pain and disorder of the visual functions.

*Photophobia and Epiphora.*

These two symptoms deserve special attention, as they invariably accompany keratitis; they are also met with in iritis and retinitis. If we consult the various works which treat of ophthalmology, we shall find that most authors look upon photophobia and epiphora as symptoms not only of keratitis and iritis, but also of conjunctivitis, or of any other inflammatory affection of the eye. Reasoning and experience both show, however, that this opinion is incorrect, and that the phenomena in question are always the result of a lesion of the cornea, the retina, or the iris, and never that of simple inflammation of the conjunctiva. Nor will it be difficult to explain how the error originated. Not being accustomed to separate the different species of ophthalmia, confounding under the same name several distinct affections of the eye, they did not perceive that the patients on whom they observed these symptoms had not only inflammation of the conjunctiva, but also inflammation of one of the organs I have just named. Now I ask you, can it be rationally allowed that an inflammatory affection of the conjunctiva—a membrane which has no connection with vision—is capable of producing photophobia? In some cases of conjunctivitis, it is true, you see the patients carefully conceal their eyes, and take every precaution to protect them from the light; but this is not owing to photophobia. If they do so, it is merely to avoid the pain which the contact of the air with the inflamed conjunctiva occasions. Some patients also keep their eyes closed, and their eyelids immoveable, because the slightest motion of these organs gives rise to the painful sensation of extraneous bodies between the eye and the eyelid, to which I have so often alluded in speaking of conjunctivitis. A circumstance which proves also that conjunctivitis is not the cause of these phenomena is, that when this affection exists alone, the patient suffers acute pain, it is true, if the eyelids are separated, but is, nevertheless, able to examine every thing around him without the slightest difficulty. On the other hand, it is extremely easy to prove that a lesion of the cornea may produce these symptoms, as the slightest abrasion of that membrane on a living animal is immediately followed by intense photophobia and epiphora.

The intensity of the photophobia varies in the different forms of keratitis. In chronic and in diffuse keratitis it is but slight; its greatest intensity is, when there is ulceration of the cornea; indeed, it is possible to form a diagnosis of the existence of ulceration in eighteen cases out of twenty, from this symptom alone, without examining the eye. Whenever you see a patient who hides his face in his hands, or in the bed-clothes—who contracts his eyelids forcibly if you attempt to open them—from whose eyes a torrent of tears gushes forth as soon as they are exposed to light—you may be certain there is more or less abrasion of the cornea existing. The contact of the air with the ulcerated surface accounts for the intolerance of light being so great.

Epiphora is even a more characteristic symptom of keratitis than photophobia, no symptom showing more clearly the difference which exists between the inflammatory affections of the cornea and those of the conjunctiva. In conjunctivitis there is a more or less abundant secretion of mucus, which collects in the inner angle of the eye; but, be the inflammation ever so great, even when chemosis exists to a considerable extent, there is no unusual secretion of tears. In keratitis, on the contrary, however violent the inflammation, there is scarcely any secretion of mucus, whilst the effusion of tears is generally so abundant, that the eyes seem literally bathed in water. This, however, only applies to the cases in which the above diseases exist singly; if they are combined, you will necessarily meet with both epiphora and a mucous secretion. The tears also appear to have acquired irritating properties, which they do not possess in the healthy state, as they give rise frequently to a scalding sensation, and produce erythema, or even slight excoriation of the surface over which they pass.

If the inflammation runs high, the pain is often severe, so much so as to render sleep impossible; this is more especially the case when the cornea is ulcerated. It generally occupies the orbit, from whence it extends to the temples—to the forehead—sometimes all over the head, and is much more violent in some persons than in others—in the evening, and during the night, than in the morning. This latter circumstance is one of the reasons which have induced ophthalmologists to think that the inflammatory affections of the cornea are of a rheumatic nature. The exacerbation of the pain during the night, ought not, however, to have led to such a conclusion, as there are other maladies—such, for instance, as secondary venereal affections—in which the same symptom is observed.

*Disorder of the Visual Functions.*

In the different forms of conjunctivitis we have only found the functions of the eye impaired, as it were, accidentally, when chemosis exists to such an extent as to encroach on the cornea, and obstruct the passage of light. This is not the case with the disease we are now examining; the slightest alteration in the tissue of the cornea invariably more or less impedes the phenomena of vision. This circumstance alone would suffice to distinguish the affections of the cornea from those of the conjunctiva, were other characters wanting.

In keratitis, vision is more or less disordered, according to the form and activity of the inflammation—according, also, to the stage at which it has arrived. If there is but a slight suffusion of the cornea, the objects which surround the patient merely appear rather dimmed; indeed, frequently no difference is perceived. When, however, the suffusion becomes greater—when it assumes the form of nebula—every thing appears as if seen through a mist, the density of which depends on the degree of opacity which the cornea presents. If there exists an ulceration, or a circumscribed collection of coagulable lymph, between the lamellæ of the organ, sight is very seriously impaired, especially when the lesion is opposite the pupil. Alteration of the tissue of the cornea is not, however, the only cause which may impair the visual functions. The aqueous humour may become affected, and assume a milky appearance; the iris may be inflamed, and the contraction of the pupil which then takes place may be so great, as to obstruct the entrance of light into the eye; the inflammation of the cornea may also be so intense as to extend to the crystalline lens, or to the vitreous humour.

Keratitis, as I have already told you, is to be met with both in the acute and in the chronic form. It is to the acute form of the disease that I shall now direct your attention.

*Acute Keratitis.*

Acute keratitis is, undoubtedly, the most common form of the disease. The inflammation may assume the chronic type at the commencement, it is true; but cases of this kind are not very frequent. Acute keratitis may be superficial, interstitial, or deep-seated. Each of these forms must be examined separately.

*Superficial keratitis.*—Inflammation of the superficial lamella of the cornea is the least serious form of the disease, and often owes its origin to conjunctivitis, in which case the characters of the two affections are more or less blended. It is also the form of inflammation which is generally produced by external violence. The following are the anatomical characters by which it may be distinguished:—The cornea at first presents a slight suffusion, which, if examined with care, will be found to reside evidently in the external lamellæ. In the course of a day or two it loses its polished appearance, and becomes covered with small granulations, which are not always perceptible to the naked eye, but may be easily seen with a magnifying glass. They may be compared, in every respect, to those which we meet with in granular blepharitis or conjunctivitis, and, like them, are probably due to the hypertrophy of the glandular organs which exist in the external membrane of the cornea. Even when the cornea is primitively affected, that portion of the conjunctiva which surrounds it soon becomes inflamed. Some anatomists deny the passage of the conjunctiva over the cornea; admitting, however, that they are right, we must conclude that the two membranes are continuous; and in either case, the connection which exists between them explains satisfactorily the rapid extension of the inflammation from one to the other. You see, therefore, that in superficial keratitis the conjunctiva is nearly always either primitively or consecutively inflamed. Its vascularity is often so great as to conceal the more deeply seated redness of the sclerotica. Numerous vessels are also frequently seen passing from it on to the cornea, under the form of delicate vascular filaments; sometimes they form a kind of triangular or semi-lunar network, the base of which rests on the conjunctiva, whilst the summit advances more or less on the cornea, and terminates by a small pustule, or by a white speck. If the inflammation increases, one or more small phlyctenæ appear, due to a rising of the external membrane of the cornea; between this and the tissue of the organ a little coagulable lymph, or even pus, is effused, thus giving rise to a small abscess. These abscesses generally open externally; but sometimes, instead of perforating the outer membrane, they extend to the inner lamellæ, and occasion the interstitial form of inflammation.

When superficial keratitis is not accompanied by inflammation of the subjacent tissues, there is but little photophobia or epiphora. The objects which surround the patients are distinguished by them, although they appear enveloped by a mist of variable density. Generally speaking, the affection is not serious, and, if properly treated, may be cured in a few days, the cornea completely recovering its transparency in the cases in which there has been no ulceration.

*Interstitial keratitis.*—The interstitial form of inflammation is the one which is the best known, and has been the oftenest described; indeed, as it is the tissue of the cornea which is inflamed, it is to this affection that the word keratitis more especially answers. Superficial keratitis may be confounded with conjunctivitis; and deep-seated or internal keratitis with iritis; but the interstitial form can scarcely be confounded with either of these affections. It is, also, to this form that what has been said respecting the general symptoms of keratitis principally applies. The anatomical characters it offers are the following:—At the commencement of the disease the cornea often presents the water-green tint I have before described, but its transparency soon becomes much more diminished, and the patient complains that he cannot distinguish the objects which are near him from one another. The cornea assumes a muddy appearance, and effusion of coagulable lymph takes place between its lamellæ, either extending over the entire membrane, or occupying a more or less circumscribed portion of its surface, and that without there being necessarily any phlyctena, any erosion or ulceration of the external membrane. The vascular zone of the sclerotica is exceedingly well marked, and presents the characters with which you are already acquainted. The vessels, advancing in a parallel manner, converge as they approach the cornea, and form a red band of variable width. When there is no ulceration, you will often see very plainly, in the tissue of the cornea, many of them advancing, under the form of small filaments, towards the centre. In many instances there is little or no inflammation of the conjunctiva.

There is but little photophobia or epiphora, unless the cornea be ulcerated; in which case, on the contrary, the intensity of these symptoms is very great. Interstitial keratitis is decidedly the most dangerous form of the disease, and deserves all the attention the surgeon can bestow.

*Deep-seated keratitis.*—In this form of keratitis the inflammation occupies that portion of Descemet's or Demour's membrane which lines the posterior surface of the cornea. Having hitherto been but little studied, and but imperfectly known, the symptoms of this affection have evidently been confounded with those of iritis: in one, the internal surface of the cornea is inflamed; in the other, the anterior surface of the iris. The reasons which have induced him to make this division are not, however, sufficiently conclusive; new researches are necessary before it can be recognised. M. Jüngken asserts that in this form of keratitis, reddish streaks, formed by injected vessels and opaque specks, may be seen on the membrane lining the posterior surface of the cornea. I have myself never seen these appearances, although I have paid great attention to the subject. Indeed, when I take into consideration the distribution of the vessels of the eye, I can scarcely understand how these phenomena could take place. The opacities which M. Jüngken and some other ophthalmologists say they have seen, may also be merely the result of an optical illusion. Reflection of light might easily, under certain circumstances, lead us to suppose that a speck occupies the posterior surface of the cornea, whilst, in reality, it is situated on the external surface of that membrane.

There are several symptoms by which this affection may be distinguished from the other forms of inflammation. The external surface, as also the tissue of the cornea, remains healthy. The water-green tint, which, as I have already stated, probably depends on an alteration in the aqueous humour, is very evident during the first period of the inflammation; the aqueous humour gradually becomes more troubled, assuming a turbid appearance, especially in the inferior portion of the anterior chamber. This may be easily explained: the dimness of the aqueous humour is owing to the effusion of coagulable lymph, which, being of greater specific density than the aqueous humour itself, naturally accumulates in the lower part of the anterior chamber. This may also be observed in iritis; but the following symptom is peculiar to the affection of which we are now treating. On examining very attentively the cornea, you will perceive on its internal surface a number of minute spots, of a grayish yellow colour. Very often it is not until the eye has been examined several times that this peculiar appearance becomes manifest. The spots, which appear as if seen through a pane of glass, are either congregated on one part of the membrane, or scattered; they may be compared to the granulations of the external surface of the cornea. Sometimes there is a collection of matter formed between the internal membrane and the tissue of the cornea, in which case the membrane may give way, and the pus fall into the anterior chamber; or the inflammation may become interstitial.

The pain is more severe in deep-seated keratitis than in any other form of the disease. It is principally felt over the orbits, and in the back of the head, and is accompanied by throbbing in the eye, and by a sensation of fulness and distention. This arises from an increase in the quantity of the aqueous humour; the sclerotica, being of a fibrous nature, cannot give way, the eye becomes distended, and an eccentric compression is established, which gives rise to the peculiarly painful sensations I have mentioned. There is less photophobia or epiphora than in superficial or interstitial keratitis; nor will you be surprised at this, if you remember what I told you in speaking of this symptom. Indeed, photophobia appears to depend principally on ulceration of the cornea, and on the contact of the air with the excoriated surface which the cornea then presents. We have an instance of the pain which this contact occasions in blisters. When the epidermis is intact, the patient suffers but little; but as soon as it is taken off, and the cutis vera is exposed, the pain becomes very acute.

I am inclined to think, from what I have seen of this affection, that it is more frequently met with than is generally supposed. If I am right, practitioners would do well to direct their attention to the study of a disease, which, as I have already stated, has been hitherto but little noticed.

From what I have said—from your own observations at the bedside of the patients, you must be now well aware that the three forms of inflammation I have described may really be met with distinct from one another. But you must not think that is always so; the cases in which they are distinct, after the first few days, may, on the contrary, be considered as exceptions to the general rule. As, however, even when combined, one form generally predominates over the others, and as the prognosis and treatment of each are different, it is indispensable that you should be acquainted with their several symptoms.

Keratitis does not always follow the regular course I have described. The more serious symptoms, instead of appearing successively, may be present nearly at the commencement of the disease. Thus, in the course of a few days, or even in twenty-four hours, the cornea may become infiltrated with coagulable lymph to such a degree as to resemble a piece of bacon fat. This disorganization of the cornea often takes place after the operation for cataract by extraction, although, before the operation, no circumstance may have existed which could lead you to anticipate such an event. It occurs, indeed, more frequently when the inflammation is the result of external than when it is the result of internal causes. It is very frequent in the different forms of purulent ophthalmia, especially in the Egyptian and in the gonorrhœal conjunctivitis. In some instances the cornea, although passing on to suppuration, may be only partially destroyed, and become vascularized with astonishing rapidity. It then presents the appearance of a turgid, red, fungous mass, bathed in pus, and half concealed by the eyelid. Mr. Wardrop, it seems, has met several cases of this kind among the troops that returned from Egypt. I have myself met with a striking instance in a man affected with extensive carbuncular inflammation of the two right eyelids. The cellular tissue of the eyelids was mortified; and when we were able to separate them, we found the cornea of a livid red colour, looking like a kind of cherry, and interspersed with white and yellow streaks.

In keratitis the inflammation is generally diffuse, occupying the entire cornea; it may, however, be partial. When this is the case, it is nearly always of a chronic nature; and the part of the membrane which is affected often presents a pustule, a phlyctena, or an ulceration. If the inflammation is limited to a portion of the membrane, and these phenomena are not observed, it follows the course I have already described.

Whatever may be the form under which keratitis appears, its progress is much slower than that of conjunctivitis. This depends not only on the nature of the disease itself, but also on its being nearly always accompanied by other inflammatory affections, such as blepharitis, iritis, choroiditis, &c.

*Termination.*—Acute keratitis usually terminates by resolution, ulceration, suppuration, or mortification. For the present I shall only speak of resolution; the other four modes of termination will be studied at a future period.

Resolution is certainly the most desirable termination of the disease; yet, even when it does take place, the functions of the eye may remain more or less impaired. When resolution commences before any infiltration has taken place between the lamellæ of the cornea, before any collection of matter has been formed, sight is restored in all its integrity. If, on the contrary, coagulable lymph has been effused in ever so small a quantity, it is rarely completely absorbed, and gives rise, consequently, to opacity of the cornea. Softening of that organ may also take place to a certain extent before resolution commences; when this is the case its surface becomes uneven, or partly depressed; and then, although the transparency of the membrane is not altered, the sight is more or less injured.

*Prognosis.*—The prognosis of this affection is generally serious; not that any fear need be entertained for the life of the patient, but because the functions of an important organ are often seriously compromised. We ought, therefore, in every case, to do our utmost to arrest the progress of the inflammation as early as possible. The prognosis is not, however, equally serious in every form of the malady. Superficial keratitis may be completely cured, providing, as I have just stated, resolution commences before any effusion has taken place. Deep-seated keratitis is a more dangerous affection, from its immediate connection with the internal parts of the eye. When improperly treated, the inflammation may invade nearly all the tissues which occupy the interior of the eye, and be followed by the total loss of that organ. The prognosis of interstitial keratitis is also more serious than that of the superficial form; it is this form of inflammation which most frequently gives rise to collections of matter, to abscesses, to hypopion, and to the more serious forms of opacity of the cornea. I would also remark to you, that the importance of the different forms of opacity depends in a great measure on its situation. Thus, a simple nephelion, or nebula, situated opposite the pupils in the axis of vision, will prove a greater impediment to sight than an albugo, or a leucoma, on another part of the cornea.—*Ibid.*

---

*Rheumatism of the Uterus in Pregnancy and Parturition.*—The three following cases, which afford some evidence in favour of the existence of this affection, are from a memoir by M. Dezeimeris, in a late number of "L'Experience."

J. D. aged 33, during her fourth pregnancy, had in consequence of a chill four weeks before the end of her time, a tense lancinating pain in the uterus, with fever. Diaphoretics diminished this pain, but it was replaced by others, which fixed sometimes in the upper, and sometimes in the lower extremities. During delivery, the uterine contractions were extremely painful, and from the first day they drew shrieks from the patient without producing the least dilatation of the orifice. The uterus could not be touched without causing most acute suffering; but bleeding and fomentations soothed these pains, and brought on true labour pains, and the accouchement was completed in a short time.

After three days the rheumatismal pain of the uterus reappeared, and required bleeding, ammoniacum, and calomel. Suddenly the pain ceased, and the disease established itself in the muscles of both fore-arms, preventing the patient from holding her infant; and they then disappeared from those parts and fixed on the left knee. All indisposition then ceased in the rest of the body, but the knee swelled and was intolerably painful. Numerous bleedings, calomel, and other means, only gradually diminished these symptoms; but weakness and stiffness of the joint remained for some time, and required the use of crutches.

Q. R. aged 24, of a strong constitution, was received pregnant with her first child in the Maison d'Accouchement of Trèves; during the greater part of her gestation she had had to wash linen in a very cold room, with damp clothes on, and her feet in the wet.

Soon after her admission, (on the 26th of October) she felt pains, which seemed the preludes of labour, succeeding each other at unequal intervals, but producing only slight dilatation of the uterus, though they were very severe. On the 27th, the pains sometimes ceased for a whole hour. On the 28th, the os uteri was more than an inch in diameter, and the membranes were distended like a bladder within it; the pains were strong and frequent, at the same time that the movements of the fœtus were active and painful; in the afternoon the pains became weaker, and the orifice was a little contracted. On the 29th, the orifice was completaly contracted, and the patient complained of a severe pain in the right iliac region, and a sensation of weakness in the whole body. On the 31st, the abdominal pain continued, with headach; the pulse, which from the commencement had always been frequent, was still so; there was not the least vestige of uterine contractions. The urine was red, and discharged with a kind of tenesmus of the bladder. The rheumatismal nature of the case was no longer doubted, and an infusion of calomel and valerian, with Hoffman's anodyne, was administered as a diaphoretic. On the 1st of November, the febrile heat and the sweat had rendered the night very restless; the headach continued, but all the abdominal pain had ceased, and the pulse had lost its frequency. On the 2d, the patient felt quite well. On the 26th and 27th the same pains of the abdomen returned again, but soon ceased, and on the 16th of December the case was terminated by a natural delivery.

D. K. aged 24, had been exposed to a great deal of cold and moisture. In the 6th month of her pregnancy she caught cold, and had rheumatic fever with cough, and tearing pain in almost every part of the body, and especially in the sacral and pubic regions. After eight days she came to the hospital with the following symptoms: stiffness in the back; lancinating pains in the chest; dry and painful cough; sense of tension in the sacrem, abdomen, and pubic region; loss of appetite; frequent febrile and small pulse. The fundus of the uterus was two fingers' breadths below the pit of the stomach; the abdomen was very sensitive on pressure. Infusion of calomel and acetate of ammonia, with hot fomentations to the abdomen, were ordered; after a copious sweat the patient was relieved, and in a few days was completely restored to health.

About a month afterwards she was delivered with the forceps. The pains in the abdomen and other parts returned after the accouchement, and continued for ten days, when they were replaced by pain and tension in the left shoulder; after three days these diminished, and the abdominal pains returned, but they were now promptly removed by antispasmodics.

*Equivocal Generation.*—Schultze and Schwann have examined some of the phenomena of equivocal generation. The former filled a glass vessel with distilled water containing various organic substances, and closed it with a cork, through which two bent glass tubes were passed; the water was then boiled, and while the vapour was rising in the tubes, a Liebig's apparatus was attached to each; that of one containing some concentrated sulphuric acid, and that of the other some concentrated solution of potash. The air could thus be easily renewed, but whatever germs there were in it would be destroyed by having to pass through the concentrated acid.—The whole apparatus was then placed at a brightly lighted window, and by its side an open vessel containing an infusion of the same organic substances: infusoria were generated in the latter, but in the former not a trace of them could be discovered.

The result of Schwann's experiments is the following:—

If a closed glass sphere, filled with atmospheric air, and containing besides a small quantity of infusion of muscular tissue, be exposed to the boiling heat of water, after several months there will be observed neither the formation of infusoria, nor any putrefaction. And it will be just the same though the air be renewed, if that air which is introduced be previously exposed to a high temperature.—*Müller's Archiv. Jahresbericht*, No. clxxvi.

[These experiments seem to settle this long disputed question. They are the first in which every possible entrance for living ova dispersed in the atmosphere has been closed, and in a case of this kind it is evident that a single negative result is worth many positive.—*Lond. Med. Gaz.*]

*Typhus Fever—the Saline Treatment.* By William Marsden, M. D.—Many cases of typhus fever having occurred during the last three weeks, among the lower orders, I cannot refrain from making known to the profession generally, the result of a mode of treatment, simple in its nature, yet founded on scientific principles, which I have adopted on many former occasions. I shall simply, in the present instance, relate the facts of the case, but at some future opportunity shall, in the event of your inserting this communication in your Journal, give my views of the philosophy of the practice.

On Sunday, September 1st, Richard Berry, aged 47, and Elizabeth Davis, aged 67, were admitted into the hospital with typhus fever, from Seacoal lane, in which neighborhood there had been eleven persons afflicted in a similar manner, five of whom had died. They had both been under treatment for some days previously to their admission. The woman died on the second day after her reception, and it was expected that the man must inevitably share the same fate. The following was the condition of the patient:—Pulse scarcely perceptible, beating upwards of 100; tongue black, thick, and dry; lips thick and black; breath fœtid; respiration short and interrupted; extremities cold; patient could take no food but liquids; evacuations involuntary; with great difficulty could be made sensible to any sound, and could not answer any question. The following mixture was forced into his stomach:—

*Muriate of soda*, one ounce;
*Oxymuriate of potash*, ten grains;
*Water*, one pint. A wineglass full given every hour.

Between each dose half a pint of cold water or weak broth given to the patient.

Four and twenty hours after administering these remedies, the patient's pulse improved; the tongue and lips changed from black to red; the breath became pure, and respiration more free and regular.

Four and twenty hours after this the patient improved still more, and was in a condition to answer questions, and also to swallow his medicine without force. After another twenty-four hours he was able to sit up, enter into conversation, and express all that he felt and desired. He was now ordered half a pint of porter every two hours, and the same saline solution every two hours, to be given alternately. Since which period he has continued to recover most rapidly, and will be able to leave the hospital, perfectly restored to health, after a few more days.

This is a mode of treatment which I have adopted in typhoid and low fevers, with similar results for some years past, and with the utmost success; and it being a plan of treatment perfectly at variance with ordinay practice, and more particularly so with that of practitioners and teachers of the old school, I am induced to submit it to the consideration of the profession generally, reserving to myself an opportunity, on some future occasion, to give my reasons for pursuing so simple a remedy, and the one which I believe to be founded on the only principle upon which such maladies can with any thing like success be treated.—*London Lancet.*

*On the Treatment of Gravel.* By M. Civiale.—The formation of gravel has generally been attributed, by those who have paid attention to urinary diseases, to the operation of chemical laws, and the means of cure have equally been directed to the destruction of the product thus formed, without any reference to the organic changes which favor their formation, or to the influence they exercise, by their simple presence, on the animal economy. Authors, in treating of calculous disorders, are too frequently in the habit of considering as the original disease that which in reality is merely an effect or result of one or more morbid states; and their chief care has been directed to discover some mode of eliminating the foreign body, without bestowing a due share of attention on the organic modifications under the influence of which it has been formed. Formerly, writers asserted that gravelly affections were confined to warm climates, and that they were most frequent amongst elderly persons addicted to good cheer. Both of these errors have been corrected by more exact observations.

The chemical theory of the formation of gravel can only apply to the period at which the urine, having passed through a series of morbid states, is disposed to the deposition of gravel. But we must ascend a step higher in the investigation. What gives rise to this morbid disposition? What is the reason that in one case we find a predominance of uric acid; in another of the urate of ammonia; in a third, of the oxalate of lime; in a fourth, of the phosphates, &c.? Such are the questions which we must be prepared to answer, if we desire to raise the treatment of calculous disorders above the level of empiricism. I have endeavored to resolve these questions, and thus discover a rational mode of treatment, the beneficial effects of which have been confirmed by extensive experience.

Almost all the cases of gravel which we find recorded in medical works are incomplete, and this necessarily depends on the manner in which they have been observed.

A patient is affected with gravel: antiphlogistics, alkalies, &c., are ordered; the symptoms are relieved, or disappear; the physician loses sight of his patient, and thinks that he is cured. But the affection returns at some distant period, and the patient seeks another medical attendant. He has now a calculus in the bladder or in the kidney. Such cases I witness every day. It is easy to explain their occurrence. The accidents occasioned by the gravel, or even by urinary calculi, are but temporary, and disappear of their own accord, after a certain lapse of time.

The main object of practitioners, in the treatment of gravel, has usually been to favor the discharge of the foreign substance through the urethra. Sometimes, in fact, we find that very large fragments of stone are passed through the canal, after lithotrity, without occasioning any pain or uneasiness. At other times a very small particle will give rise to the most agonizing sufferings. This difference depends on the manner in which the patient has been prepared for the operation, by the employment of means calculated to diminish the sensibility of the urethra, &c.; and, hence, it was natural to employ similar measures in the treatment of persons affected with gravel. A variety of morbid conditions oppose the spontaneous discharge of gravelly concretions; the chief are spasmodic or organic contraction of the urethra; diseases of the prostate and neck of the bladder, and atony, or paralysis of the bladder. In a patient not affected with gravel, these complications will render the discharge of urine slow and painful; with how much greater energy must they oppose the exit of a foreign body? I have treated a great many patients laboring under gravel, who had before tried, without success, all kinds of remedies. On examining the urethra I frequently found that the canal was in a state of spasmodic contraction, which had entirely destroyed its natural pliability. On removing this morbid state of the urethra, the calculous concretions were discharged with facility, although no internal treatment had been employed. In slighter cases the gravel is expelled notwithstanding the spasmodic or nervous affection of the urethra, but its expulsion is attended with considerable pain and difficulty. This state is also speedily corrected by means calculated to remove the spasm or neuralgia of the urinary canal. These results I have obtained in a great number of cases where strict vegetable diet, enormous quantities of the alkalies, various mineral waters, &c., had been employed without advantage.

Stricture of the urethra is, it is well known, a not unfrequent affection; hence an obstacle to the free passage of the calculous concretion. When the stricture is of old standing we discover its existence by various symptoms, especially the difficulty of making water; but in more recent cases the patient is able to make water well enough, although the canal is sufficiently contracted to impede the discharge of a small concretion, which is only effected by violent contraction of the bladder, and with great pain. As soon as the existence of such a stricture is ascertained, it must be attacked by the ordinary means, and then the passage of the gravel is no longer impeded. But should the stricture be more advanced, the case becomes one of very great difficulty, and requires on the part of the sufferer extreme patience, on that of the surgeon great dexterity and experience.

The prostate is subject to a variety of diseases, most of which have the effect of changing the direction of the urethra, and diminishing its natural pliability. Hence, the urinary concretions are, in such cases, usually retained in the bladder, and increase in proportion to the length of time which they are retained, and the tendency of the urine to deposit. Unless the surgeon be careful to remove this state of the prostate gland, or neutralize its influence, all medical treatment must be unavailing. Independent of the direct obstacle produced by organic disease of the prostate, or any tumor near the neck of the bladder, there is one which I would term *vital*, and which depends on increased sensibility and contractility of the urethra and neck of the bladder. This morbid sensibility is itself connected with some commencing disease of the prostate, or with urinary concretions; and the surgeon has thus to combat two distinct states, each of which acts on and augments the other.

The treatment which I have pointed out in cases of urethral neuralgia usually suffices here, but as the organic disease advances the contraction of the neck of the bladder persists, and concretions are retained. Under such circumstances I have recourse to lithotrity, for the removal of the concretions, after which the spasmodic condition of the urethra is combatted with greater success.

It sometimes happens that while the neck of the bladder is in a state of morbid contraction the body of the organ has lost more or less of its contractile power. This condition of parts is easily detected, yet we find nothing relating to it in works on the diseases of the urinary organs. In slight cases of this kind the irritability of the neck may be appeased by frequently passing a bougie, while the tone of the bladder is, at the same time, restored by injections of cold water.

But the various lesions just noticed not only impede the expulsion of urinary concretions, but also react on the kidneys in such a manner as to become a powerful exciting cause of gravel. Hence, the treatment which I have indicated both diminishes the accidents and inconveniecies of gravel, and contributes powerfully to prevent its formation.—*London, from French Lancet*, July, 1839.

---

*Formula for the Internal Administration of Turpentine.*

℞. Olei terebinth. ʒiss—ij.

Magnesiæ carbonat. ʒj.—tere simel et adde,

Aquæ menth. sat. ℥v.

Syrupi limonum ʒij.

Spir. lavend. comp. ʒij.

Misce, sumatur pars quarta ter die.—*Med. Chirurg. Rev., from Schmidt's Jahrbucher.*

---

*Useful Application to Chilblains.*—The following application is strongly recommended to relieve this troublesome affection:

Take of Balsam of fioraventi ʒij.

Solution of acetate of lead ʒiij.

Olive oil ʒiij.

Hydrochloric acid ʒj.

Shake them well together.

The affected parts are to be rubbed once or twice a day with this embrocation; and a piece of silk paper, moistened with it, should be kept constantly applied. The strength of the embrocation may be easily increased or diminished by varying the quantity of olive oil used in preparing it. When the chilblained skin has become chapped and ulcerated, the embrocation is to be applied only to the surrounding skin, and the little wounds should be dressed with laudanized cerate, to which we may sometimes add with advantage a portion of tincture of benzoin.—*Ibid, from Bulletin General de Therapeutique.*

---

*Treatment of Amaurosis by the Cauterization of the Cornea, and by Strychnine.*—M. Lisfranc has for a number of years been in the habit of treating many cases of amaurosis and of mydriasis by the application of the nitrate of silver to the cornea, with the view of stimulating the nervous and vascular apparatus of the eye, and more especially the branches of the fifth pair of nerves.

The stick of caustic should be lightly applied to five or six points in the circumference of the cornea: a slight injection of the blood-vessels of the eye is induced, but no inflammation is excited. A copious secretion of tears and of the nasal mucus follows; a smart pain is not unfrequently felt in the forehead and cheek; the pupil is contracted, and the retina becomes more impressionable, and the globe of the eye is powerfully rolled about by the spasmodic action of its muscles. In some patients the stomach is so much disturbed, that vomiting follows the application of the caustic. The following case will show the good effects of the treatment now recommended.

A man, thirty-eight years of age, had for about six months found his vision to be becoming gradually more and more indistinct, when he applied for admission into the hôpital de la Pitié. All objects seemed to be enveloped in a thick mist, and occasionally to be surrounded with luminous circles. He had been treated with blisters over the forehead and temples for several weeks; but this treatment had not produced any benefit.

When admitted into the hospital, he was so blind as not to be able to guide himself; still he could distinguish between darkness and light; the pupils were much dilated, and did not contract. M. Lisfranc cauterised the ocular conjunctiva, at the inferior part of the cornea, for about two lines and a half, on the 23d of October.

On the following day, the conjunctivæ were highly vascular; the patient complained of severe headach, and he was also slightly feverish. He was therefore bled and put on spare diet. The febrile symptoms were relieved; and, on examining the eye, the pupil was observed to be less dilated than it had been.

On the 28th, the conjunctival inflammation is reported to be less; the pupils are contracted, and the patient begins to see a little more clearly.

On the 30th, the ophthalmia had almost disappeared, and the pupils *reviennent sur elles-memes;* the patient was able to distinguish the number of fingers held before his eyes. In the course of two or three weeks, the patient left the hospital, having his vision greatly improved.—*Bulletin General de Therapeutique.*

M. Malgaigne, one of the surgeons of La Charité hospital, has in one of the late numbers of the *Lançette Française*, published several cases of obstinate amaurosis successfully treated with strychnine. From an eighth to a half of a grain is to be sprinkled on a recently-blistered surface in the neighbourhood of the affected part, as the temple or forehead. Care should be taken that the blistered surface does not become coated with a layer of lymph, as the absorption of the medicine will thus be completely prevented. Indeed, unless the strychnine produce its peculiar physiological effects—such as pricking or darting pains in the part, sparks of fire before the eyes, headach, &c., we cannot expect any benefit from its use in amaurosis.—*Med. Chirurg. Rev.*

